# Case report: Perilymphatic fistula from a round window microfissure

**DOI:** 10.3389/fneur.2023.1281023

**Published:** 2023-09-28

**Authors:** Toru Seo, Arata Kemmochi, Yosuke Koike, Mizuho Aomi, Tatsuya Shinohe, Manabu Komori

**Affiliations:** ^1^Department of Otolaryngology, Yokohama Seibu Hospital, St. Marianna University School of Medicine, Yokohama, Japan; ^2^Department of Otolaryngology, St. Marianna University School of Medicine, Kawasaki, Japan

**Keywords:** microfissure, round window niche, hearing loss, exploratory tympanotomy, perilymphatic fistula

## Abstract

A microfissure near the round window niche is an anatomical structure that communicates between middle ear and the ampulla of the posterior semicircular canal. It has been suggested that the microfissure can cause inner ear symptoms; however, the etiology has not yet been confirmed clinically. We report, to our knowledge, the first case of microfissure with complaint of hearing loss and vertigo and improvement in hearing after surgical sealing of the microfissure. A 50-year-old man complained of hearing disturbance, tinnitus with flowing-water sound in the left ear, and a floating sensation upon pushing the left tragus. He had moderate sensorineural hearing loss (43.3 dB) in the left ear for 3 days. His hearing worsened and he complained of severe vertigo. An exploratory tympanotomy was performed 8 days after onset. A microfissure and accumulation of clear fluid in the floor of the round window niche were detected, and leakage point was packed with connective tissue. One month after surgery, his hearing (20.0 dB) and disequilibrium had improved. The inner ear symptoms improved after the surgery in this case, suggesting that the microfissure might have caused the symptoms.

## Introduction

Perilymphatic fistula (PLF) is a distinctive otological disorder. It involves an abnormal opening between the inner ear and the external surface of the labyrinthine capsule, allowing perilymph to leak and causing specific disruptions in hearing, balance, or both (1–3). There are various causes for PLF. Japanese researchers have categorized it into four main categories (4): Category 1: Linked to trauma, middle and inner ear disease, middle and/or inner ear surgery. Category 2: Linked to barotrauma caused by antecedent events of external origin (such as flying or driving). Category 3: Linked to barotrauma caused by antecedent events of internal origin (such as straining, sneezing, or coughing). Category 4: Has no apparent antecedent event. Among these categories, Category 4, idiopathic, remains a subject of controversy.

The two recognized sites for PLFs from anatomical microfissure are the fissula ante fenestram at the oval window (5), the usual site of origin of otosclerosis, and a fissure in the round window niche (2, 3). A round window fissure communicates with the posterior semicircular canal (6, 7). Kohut et al. (8) claimed a relationship between the fissure and hearing and vestibular symptoms, but Shazly et al. (9) could not find a relationship between the fissure and sudden sensorineural hearing loss in 34 temporal bones (9). To our knowledge only three cases with hearing disturbances have been surgically as having a round window fissure (10, 11). In these, the fluid leakage was sealed. However, the patients did not have any measurable improvement in their hearing, so a cause and effect was not established. In this paper we present a novel case of a surgically repaired round window fissure resulting in a significant postoperative improvement in hearing.

## Case report

**Figure 1:**
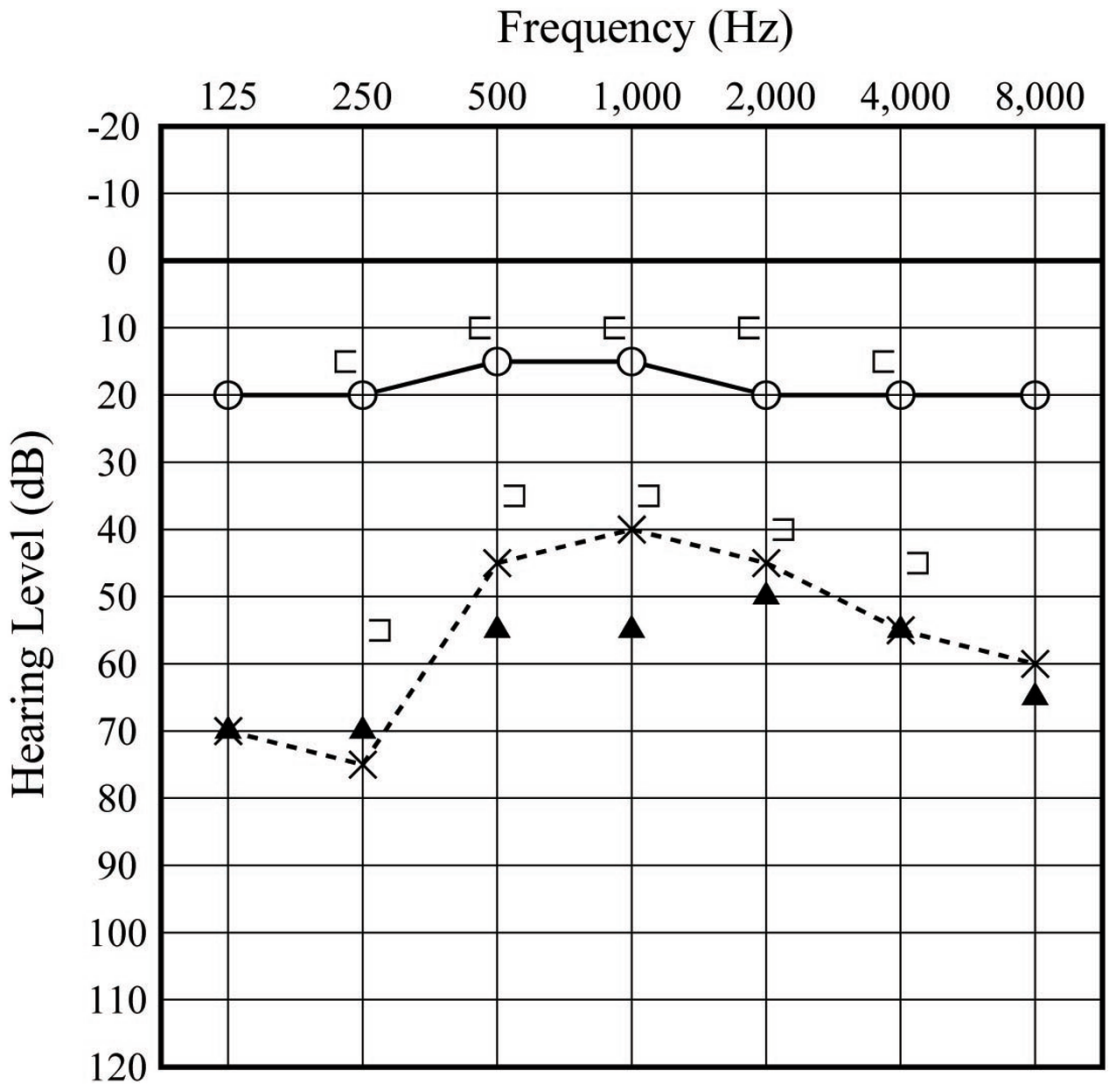
Preoperative audiogram. The open circles and x-marks indicate audiograms of the right and left ear, respectively, at first visit. Filled triangles indicate the audiogram of the left ear acquired 4 days later.

**Figure 2:**
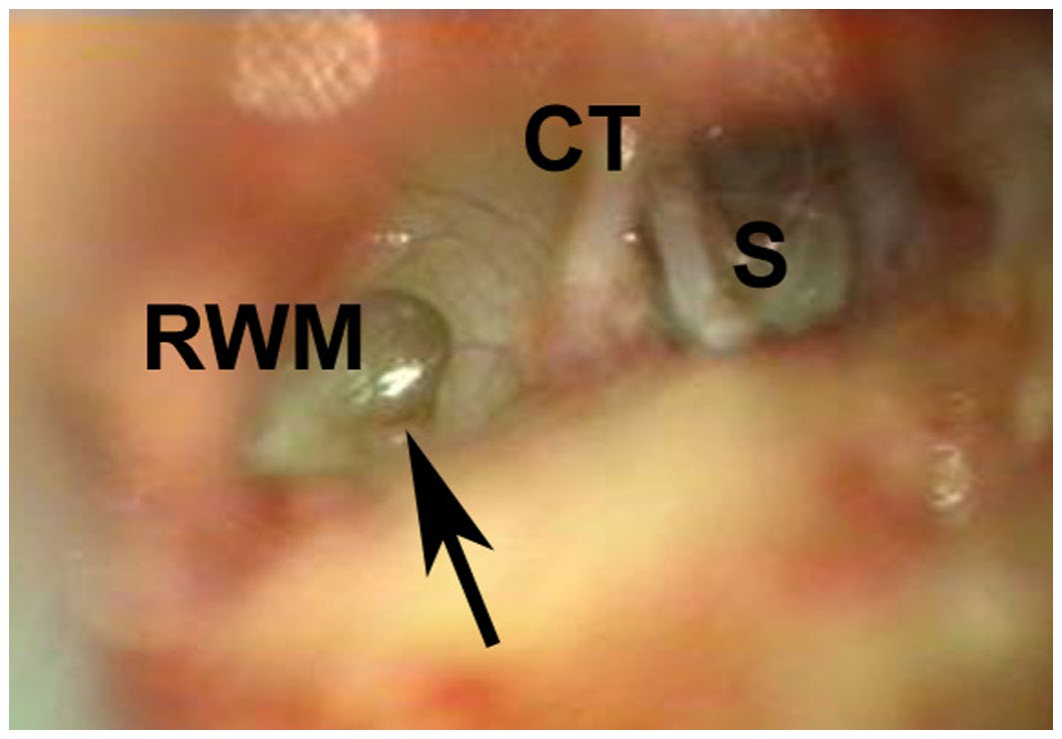
Surgical view. A small fissure (arrow) is seen in the floor of the round window niche, and clear fluid is seen leaking. CT, chorda tympani; RWM, round window membrane; S, stapes.

**Figure 3:**
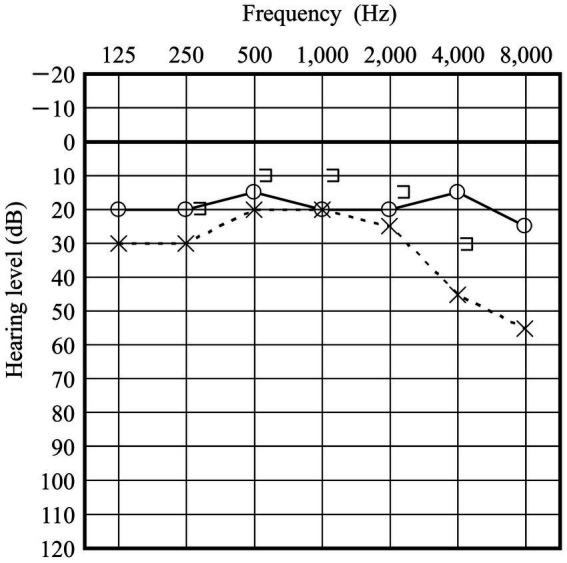
Audiogram acquired 1 month postoperatively. Hearing acuity of the left ear is improved.

A 50-year-old man presented to our clinic with a 3-day history of hearing disturbance and tinnitus with flowing-water sounds in the left ear. The symptoms did not have an instantaneous onset, but were slowly progressive. He did not remember any antecedent event related to barotrauma. He had no history of trauma, ear disease or ear surgery. Additionally, he described a feeling of floating when he pressed the tragus of his left ear. An audiogram showed moderate sensorineural hearing loss (43.3 dB) in the left ear (Figure 1). He did not have any gaze, spontaneous, or positional nystagmus. Caloric testing did not show any unilateral vestibular weakness. We strongly suspected the presence of a PLF and instructed the patient to maintain a head-up position of 30° and intravenous administration of prednisolone of 60 mg per day was initiated. Despite of these treatment, his hearing worsened (Figure 1) and he experienced vertigo with rightward beating nystagmus on lying in the left-side down position. An exploratory tympanotomy was performed on the 8 days after onset. A small fissure with accumulating clear fluid at the floor of the round window niche was observed (Figure 2). Although the fluid was absorbed carefully using a micro cotton ball, it reappeared within minutes. No fluid was observed leaking from any visible area, including near the oval window. The leakage point was packed with small pieces of connective tissue and fixed using fibrin glue. Postoperatively, his hearing (20.0 dB at 1 month after surgery) and disequilibrium improved (Figure 3).

## Discussion

In regard to the pathogenesis of the round window fissure, Okano et al. (6) found it in 100% of human temporal bones after the age of 6 years, and regarded it as a normal anatomical structure. After studying 1,000 temporal bones, Moreano et al. (7) suggested the fissure could allow bacteria and ototoxic drops to enter the inner ear, and acknowledged it had been postulated as being a potential route for a perilymph leak. Kohut et.al (8) conducted a single-blind study of 65 temporal bones to explore the connection between microfissure patency and hearing and/or balance symptoms. 86% of symptomatic patients had a patent microfissure, and 94% without symptoms had a closed microfissure. Seo et al. (12) has suggested that hearing and balance symptoms from a microfissure could be related to reduced patency of the cochlear aqueduct. Although a round window microfissure seems to be a normal anatomical structure it can be symptomatic in certain specific circumstances.

As shown in Figure 2, a microfissure was located in the floor of the round window niche in our case. Although a three-dimensional reconstruction of temporal bone study by Sato et al. (13) showed most round window microfissures are posteromediosuperior of round window niche they can be inferior as in our case.

During surgery it was challenging to determine whether the fluid observed was perilymph or exudate. Because the fluid was not tested for cochlin tomoprotein (14), we cannot directly prove that the fluid identified intraoperatively is perilymph. However, the postoperative improvement in the patient’s vestibular symptoms and recovery of hearing confirms that the symptoms were caused by the fissure. Therefore, it can be strongly inferred that the fluid was perilymph.

PLF commonly links trauma, barotrauma, ear disease or ear surgery (3, 4). The existence of cases without a history of disease, i.e., idiopathic PLF, had been controversial. In a Japan nationwide study, 19% of cases with no antecedent cause were positive cochlin tomoprotein (4), so idiopathic PLF does exist. So, what is the cause of perilymph leakage in such cases? Terayama suggests that patients with idiopathic PLF may not be aware of the cause (15). Straining, sneezing, or coughing, which trigger the onset of PLF, are events that often occur in daily life, so if the slowly progressive cases like in our case, the patient may not remember them.

## Data availability statement

The original contributions presented in the study are included in the article/supplementary material, further inquiries can be directed to the corresponding author.

## Ethics statement

Written informed consent was obtained from the individual(s) for the publication of any potentially identifiable images or data included in this article.

## Author contributions

TSe: Conceptualization, Data curation, Writing – original draft. AK: Data curation, Writing – original draft. YK: Data curation, Writing – original draft. MA: Data curation, Writing – original draft. TSh: Writing – review & editing. MK: Writing – review & editing.

## Funding

The author(s) declare that no financial support was received for the research, authorship, and/or publication of this article.

## Conflict of interest

The authors declare that the research was conducted in the absence of any commercial or financial relationships that could be construed as a potential conflict of interest.

## Publisher’s note

All claims expressed in this article are solely those of the authors and do not necessarily represent those of their affiliated organizations, or those of the publisher, the editors and the reviewers. Any product that may be evaluated in this article, or claim that may be made by its manufacturer, is not guaranteed or endorsed by the publisher.
